# Laser flare photometry: a cost-effective method for early detection of endophthalmitis after intravitreal injection of anti-VEGF agents

**DOI:** 10.1186/s12348-018-0165-4

**Published:** 2018-12-04

**Authors:** Vânia Lages, Béatrice Gehrig, Carl P. Herbort

**Affiliations:** 1 Inflammatory and Retinal Eye Diseases, Centre for Ophthalmic Specialised Care (COS) at Montchoisi Teaching Center, Rue Charles-Monnard 6, 1003 Lausanne, Switzerland; 2 Centro Hospitalar do Porto, Largo do Prof. Abel Salazar, 4099-001 Porto, Portugal; 3 University of Lausanne, Lausanne, Switzerland

**Keywords:** Laser flare photometry, Intravitreal injection, Endophthalmitis

## Abstract

**Background:**

Intravitreal injection of anti-vascular endothelial growth factor agents is the most common intraocular procedure worldwide, inevitably causing more cases of post-injection endophthalmitis. The purpose of this study was to evaluate the utility of laser flare photometry in monitoring inflammation after intravitreal injection of anti-vascular endothelial growth factor agents, particularly to detect early stage post-injection endophthalmitis.

**Main body of the abstract:**

A retrospective case review was performed of all patients who underwent flare assessment by laser flare photometry before and after intravitreal injection of bevacizumab or aflibercept at the Centre for Ophthalmic Specialized Care in Lausanne, Switzerland, between January 2015 and May 2018. The following data were retrieved: indication for intravitreal injection, medication administered, pre-injection and 72-h post-injection laser flare photometry values, and occurrence of post-injection endophthalmitis. A total of 736 injections were included in this study; 705 cases (95.8%) had a post-injection flare at 72 h ≤ 30 ph/ms, 29 cases (3.9%) had a post-injection flare at 72 h between > 30 and 50 ph/ms, and 2 cases (0.3%) had a post-injection flare at 72 h above > 50 ph/ms (664 and 742 ph/ms). These latter two cases were diagnosed as early-stage endophthalmitis.

**Conclusion:**

Laser flare photometry is a cost-effective method of screening for early stage post-injection endophthalmitis. Values > 50 ph/ms 72-h post-injection should prompt immediate evaluation by an ophthalmologist.

## Background

Intravitreal injection (IVI) of anti-vascular endothelial growth factor (VEGF) agents has revolutionized the treatment of many retinal diseases, including age-related macular degeneration, diabetic macular edema, retinal vein occlusion, and myopic choroidal neovascularization, and became the most common intraocular procedure worldwide [1]. As the number of IVIs performed has increased exponentially in the last 10 years, this has inevitably caused more cases of post-IVI infection [2]. The most recently reported incidence rates of endophthalmitis after IVI of anti-VEGF agents range from 0.013 to 0.131% [3–6]. The interval between IVI and endophthalmitis presentation is approximately 3 days [2, 7]. Therefore, optimal care means that every patient should be observed 72 h post-IVI to achieve early diagnosis and immediate treatment of post-IVI endophthalmitis. However, this scenario is impractical for many centers due to the high volume of patients receiving intravitreal treatment.

Several reports have been published on monitoring pre- and post-injection flare using laser flare photometry (LFP) [8–10]. Developed by Sawa et al. [11] in 1988, LFP is based on the same principle as slit-lamp flare evaluation, measuring back-scattered light from protein particles in the anterior chamber. The light source in LFP is a laser beam with constant energy, and the detection of back-scattered light (photons) is achieved in an automated fashion using a photomultiplier and photodetector, which make it a precise and objective device for quantifying aqueous proteins [11]. LFP allows non-invasive, objective, and quantitative measurement of intraocular inflammation, making it possible to identify abnormal increases in post-injection flare. The learning curve for the technique is short and the assessment easily performed by an ophthalmic assistant.

The aims of the present study were to evaluate mean LFP flare values pre- and post-IVI and to demonstrate the exquisite sensitivity of LFP in detecting the early stages of endophthalmitis, proposing the cost-effective use of LFP to monitor post-injection flare and uncover abnormal inflammation. The incentive to perform this study was the detection of very early signs of endophthalmitis by LFP in two cases, leading to rapid and successful management.

## Patients and methods

This was a single-center, retrospective case review performed in accordance with the Declaration of Helsinki. First, we collected the total number of IVIs administered at the Centre for Ophthalmic Specialized Care (Lausanne, Switzerland) in order to determine the total cases of post-IVI endophthalmitis between January 2006 and May 2018. Second, in order to establish the mean pre- and post-IVI LFP flare, we arbitrarily analyzed the IVIs from January 2015 to May 2018 and reviewed the files of all patients who underwent flare assessment by LFP before and after IVI of bevacizumab (Avastin; Roche AG, Basel, Switzerland) and aflibercept (Eylea; Bayer AG, Basel, Switzerland). Institutional Review Board ruled that approval was not required for the study.

The following data were retrieved: indication for anti-VEGF IVI, anti-VEGF administered, pre-IVI (same day or previous 7 days) and 72-h post-IVI LFP flare, and occurrence of post-IVI endophthalmitis. Anterior chamber LFP was performed using the FM-700 laser flare meter (Kowa Co, Tokyo, Japan). Readings were obtained by a single experienced technician (BG). Every LFP examination included ten measurements. The two highest and two lowest measurements were discarded. A single averaged reading was produced by the instrument.

IVI was performed by the same physician (CPH) in the same operating room for all patients under sterile conditions. During the procedure, the physician wore sterile gloves, surgical mask, and cap. Sterile preservative-free anesthetic drops with 1% tetracaine were placed in the eye and a periocular scrub performed using 10% polyvidone iodine, followed by a drop of 5% polyvidone placed on the ocular surface before the injection. A surgical drape covering the patient’s nose and mouth and a sterile lid speculum were used. An IVI of 1.25 mg in 0.05 ml of bevacizumab or 2 mg in 0.05 ml of aflibercept was performed 3.5 mm posterior to the limbus in pseudophakic eyes and 4.0 mm posterior to the limbus in phakic eyes. A drop of a combination of 5 mg/ml chloramphenicol and 1 mg/ml dexamethasone was administered pre and post-injection, and then prescribed three times per day for 3 days to every patient.

Descriptive and statistical analyses were obtained using SPSS® version 22 (SPSS Inc., Chicago, Illinois, USA). Continuous variables were presented as mean and standard deviation (SD). Categorical variables were presented as frequencies and percentages. The variable normal distribution was tested with Kolmogorov-Smirnov and Shapiro-Wilk tests. Wilcoxon and Mann-Whitney tests were utilized. A *p* value < 0.05 was considered significant.

## Results

Between January 2006 and May 2018, 2804 IVIs were administered at COS. Two of the cases developed bacterial endophthalmitis during this period, which gives a post-IVI endophthalmitis rate of 0.07%.

In order to determine the evolution of LFP flare from pre-injection to post-injection values, a total of 736 IVIs between January 2015 and May 2018 were considered in this study. The main indications are given in Table 1.

**Table 1:** Sample characterization of 736 intravitreal injections (IVIs)

	*N* (%)
Drug injected	
Bevacizumab	113 (15.4%)
Aflibercept	623 (84.6%)
Indications for IVI	
Age-related macular degeneration	469 (63.7%)
Retinal venous occlusion	193 (26.2%)
Diabetic macular edema	55 (7.5%)
Telangiectasia	5 (0.7%)
Myopic choroidal neovascular membrane	2 (0.3%)
Proliferative retinopathy due to central arterial retinal occlusion	2 (0.3%)
Other	10 (1.4%)

Pre-IVI mean LFP flare was similar between the two drugs, but the post-IVI mean LFP flare was higher in patients injected with aflibercept than those injected with bevacizumab (Table 2). The analysis of LFP assessment is described in Table 2.

**Table 2:** Laser flare photometry assessment

	Pre-IVI flare		Post-IVI flare		*P* ^b^
	(mean ± SD)		(mean ± SD)		
Global	11.5 ± 5.6		18.3 ± 36.4		< 0.001
		*P* ^a^		*P* ^a^	
Bevacizumab IVIs	10.8 ± 4.7	0.245	14.2 ± 5.5	< 0.001	< 0.001
Aflibercept IVIs	11.7 ± 5.8		19.0 ± 39.4		< 0.001

^a^Mann-Whitney test

^b^Wilcoxon test

**Fig. 1:**
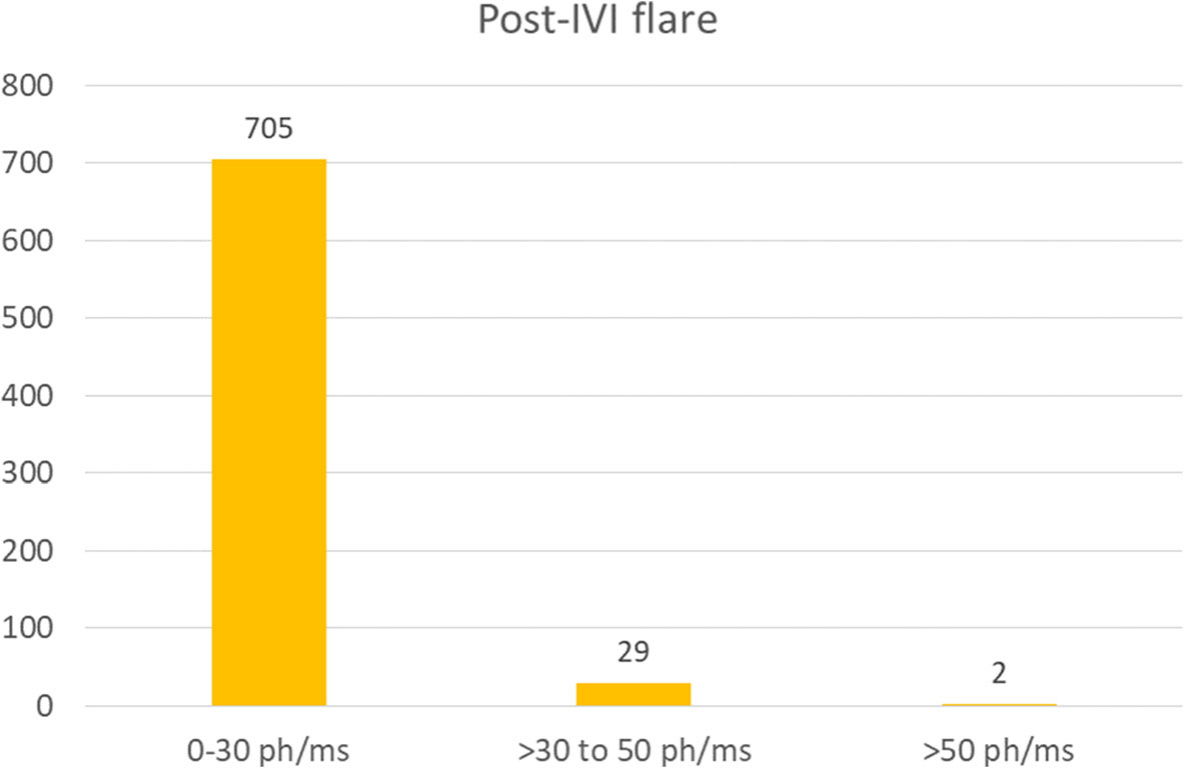
Distribution of laser flare photometry values after 72 h of intravitreal injection

Figure 1 shows the distribution of 72 h post-IVI LFP flares. A total of 705 cases (95.8%) had values ≤ 30 ph/ms (range 3.3 to 29.6*).* Twenty-nine cases (3.9%) had values between > 30 and 50 ph/ms (range 30.2 to 50.0). These patients were asymptomatic and clinical examination revealed 1+ or less anterior chamber cells. The frequency of administration of the topical combination of antibiotic and steroid was increased and more frequent follow-up visits where scheduled. No complications were detected and LFP flare returned to pre-injection values. Two cases (0.3%) had values > 50 ph/ms, namely 664 and 742 ph/ms, and were diagnosed with early-stage endophthalmitis (see case reports below).

### Case 1

**Fig. 2:**
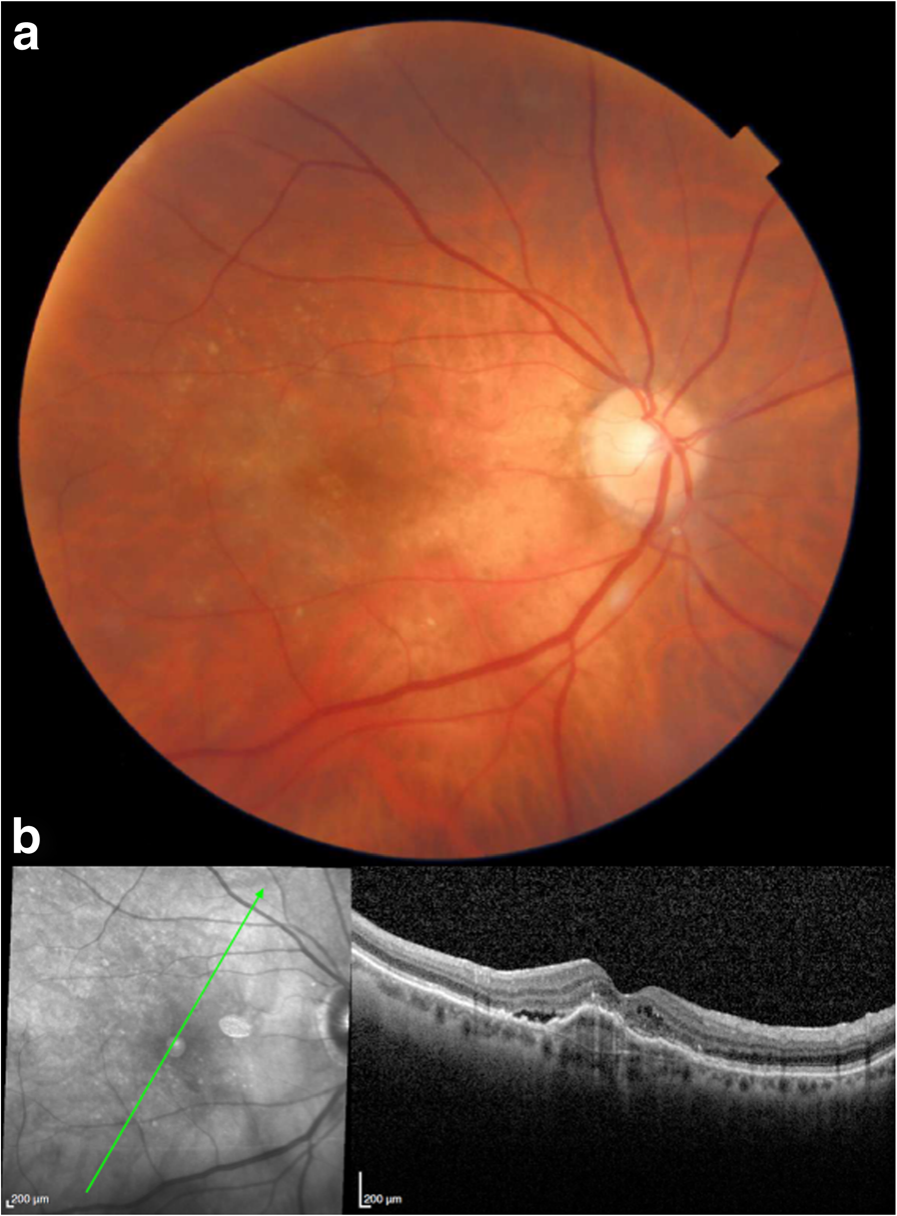
Patient 1. **a** Color retinography showing drusen and **b** OCT showing fibrovascular pigment epithelium detachment with subretinal and intraretinal fluid

**Fig. 3:**
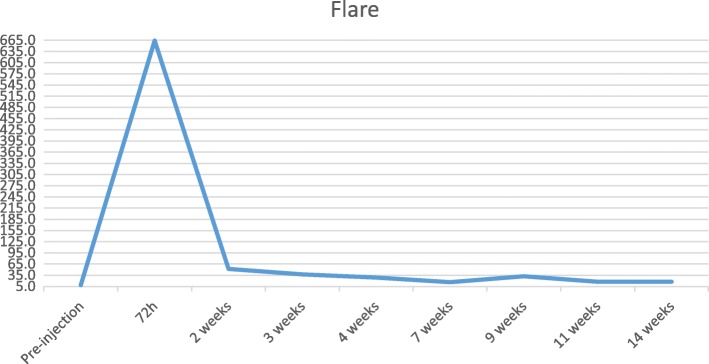
Patient 1. Laser flare photometry values evolution from pre-injection level until last follow-up

**Fig. 4:**
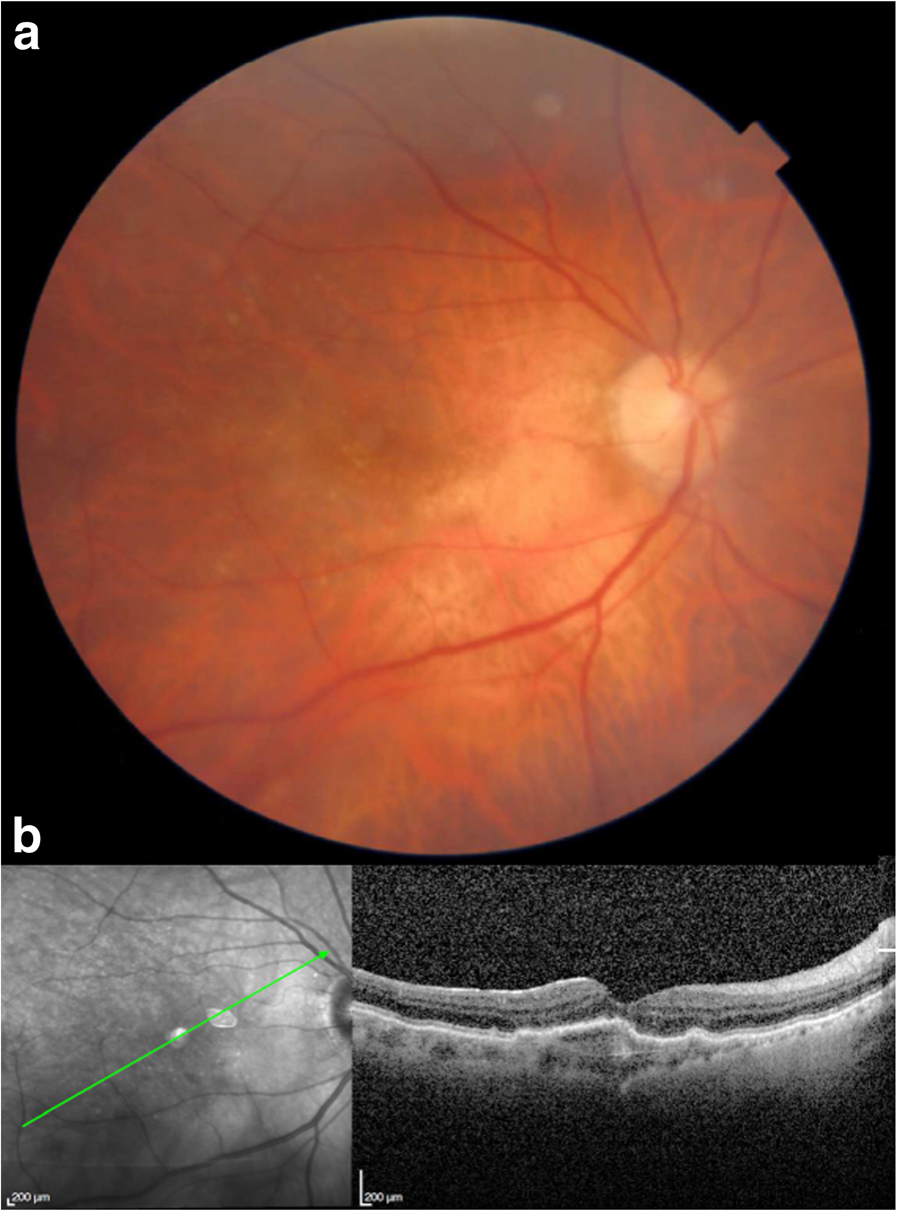
Patient 1. **a** Color retinography showing drusen and **b** OCT showing fibrovascular pigment epithelium detachment with resolution of subretinal and intraretinal fluid

A 78-year-old female had bilateral exudative age-related macular degeneration (AMD) diagnosed in October 2015 and was under treatment with bilateral intravitreal anti-VEGF injections. In February 2018, the best corrected visual acuity (BCVA) of her right eye had decreased from 0.4 to 0.2, and the optical coherence tomography (OCT) revealed recurrence of macular exudation (Fig. 2). The patient was treated with an IVI of aflibercept. At the 72 h post-IVI control visit, the patient was symptomless. The BCVA of her right eye had improved to 0.3 and no macular fluid was present. However, the LFP flare had increased from 8.6 ph/ms pre-injection to 664 ph/ms. Clinical examination revealed discreet conjunctival hyperemia, clear cornea, 3+ cells in the anterior chamber, and no hypopyon. On fundoscopy, the vitreous was slightly cloudy. Because of the tremendously high flare and first signs of endophthalmitis, the patient was immediately referred to the retinal department with suspicion of post-IVI endophthalmitis. After 2 h, before vitrectomy was performed, the BCVA was counting fingers, biomicroscopy showed a fine hypopyon in the anterior chamber, and the retina could not be visualized on fundoscopy. A diagnostic vitrectomy was performed followed by IVI of 0.1 ml vancomycin and 0.1 ml ceftazidime. At the same time, systemic moxifloxacin (400 mg/day) was started with daily parabulbar injections of betamethasone for 7 days. Microbiological analysis identified *Staphylococcus epidermidis*. The treatment response was satisfactory with gradual flare regression (Fig. 3). At the last follow-up visit 4 months later, the patient had a BCVA of 0.3, a flare of 17.9 ph/ms, and OCT showed a dry macula (Fig. 4).

### Case 2

**Fig. 5:**
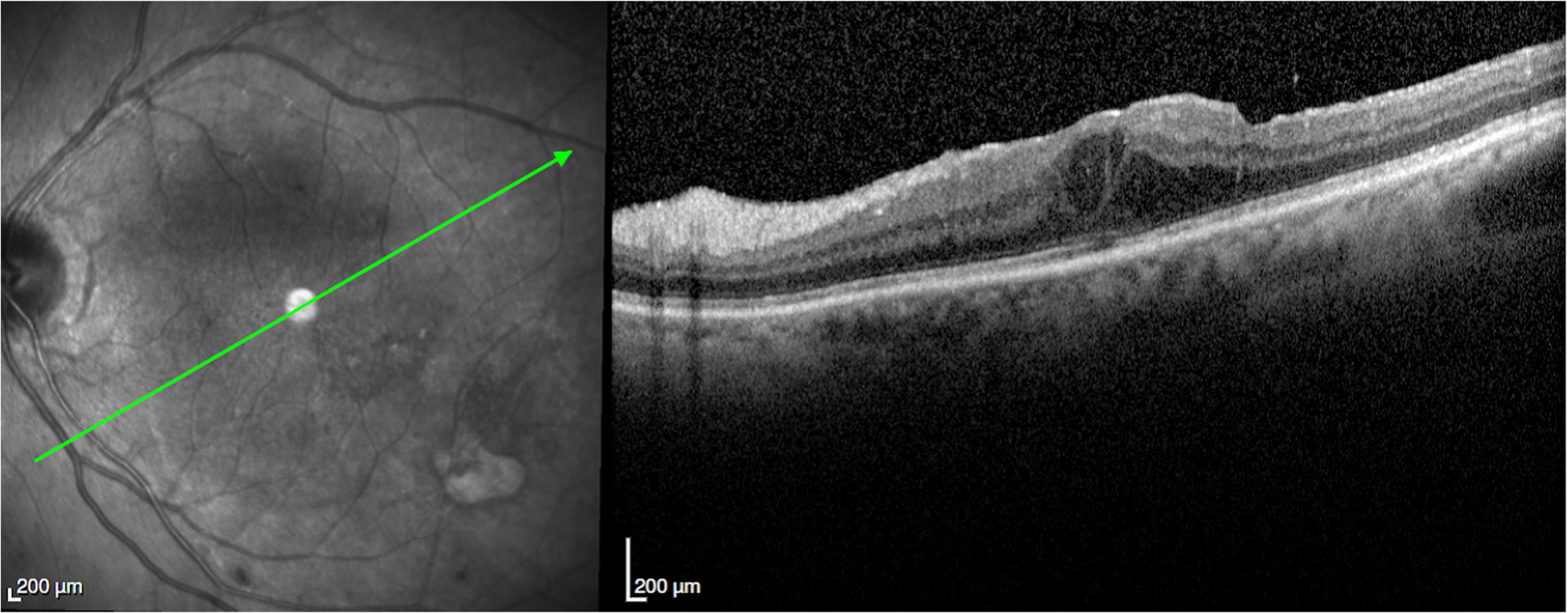
Patient 2. Pre-injection OCT showing intraretinal fluid due to diabetic macular edema

**Fig. 6:**
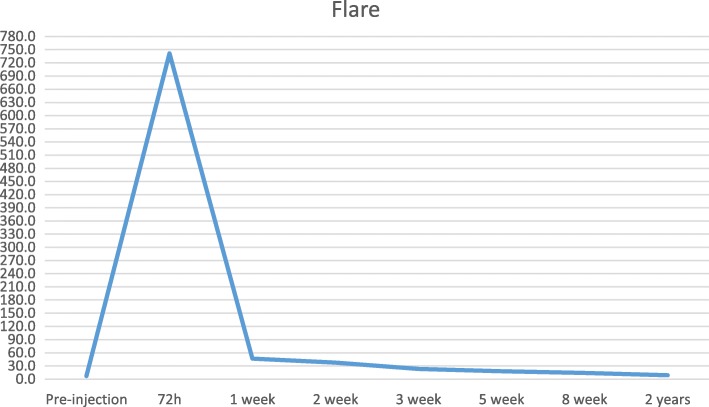
Patient 2. Laser flare photometry values evolution from pre-injection values until the last follow-up

**Fig. 7:**
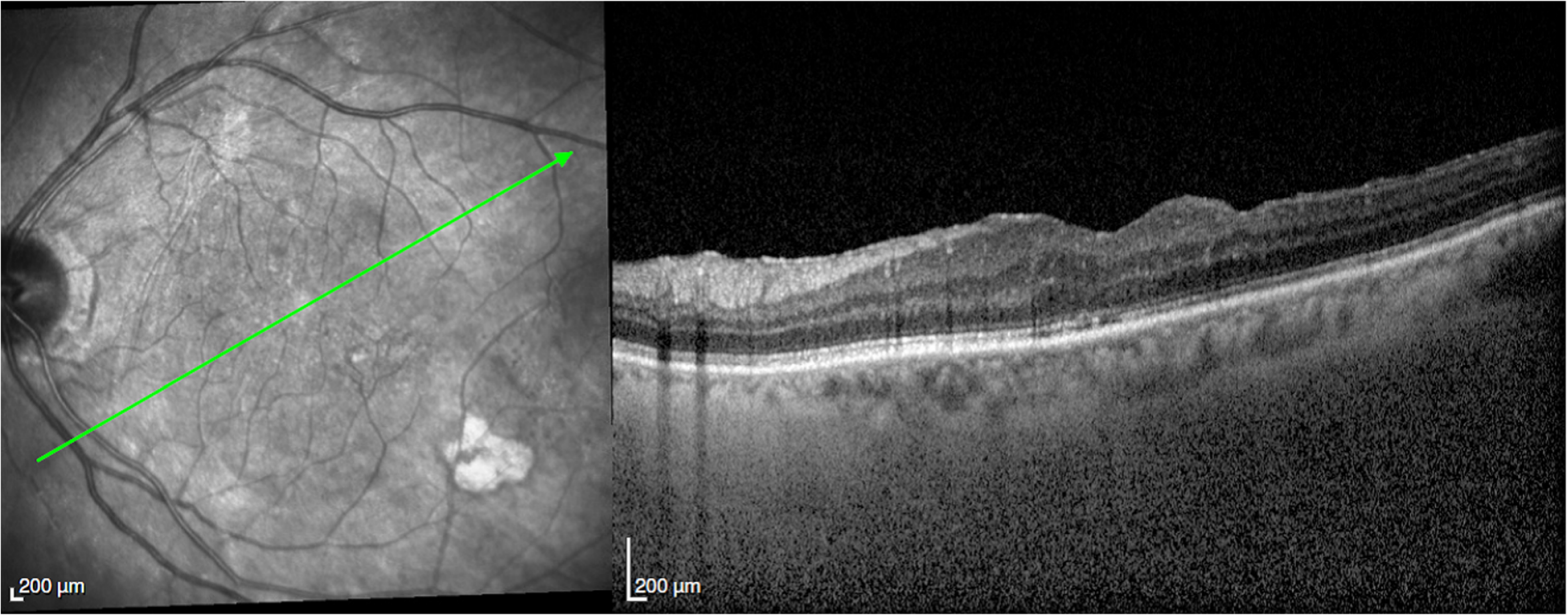
Patient 2. OCT at the last follow-up visit with decreased intraretinal fluid

A 72-year-old female with bilateral, moderate, non-proliferative diabetic retinopathy had a BCVA in the left eye of 0.7 in October 2015. The OCT revealed diabetic macular edema (Fig. 5). She was treated with an IVI of aflibercept. At the 72-h post-injection control visit, the BCVA was 0.5 and the LFP flare had increased from 7.0 ph/ms pre-injection to 300.4 ph/ms. The patient was treated with a combination of 0.5% chloramphenicol and 0.1% dexamethasone drops every 10 min for 2 h. The LFP flare was assessed again and had further increased to 741.9 ph/ms. Clinical examination revealed conjunctival hyperemia, a clear cornea, 3+ cells in the anterior chamber without a hypopyon, and 3+ vitritis. The patient was then immediately referred to the retinal department with suspicion of post-IVI endophthalmitis. During her stay in the emergency room, a hypopyon formed. A diagnostic vitrectomy was performed with IVI of 0.1 ml vancomycin and 0.1 ml ceftazidime. At the same time, systemic moxifloxacin (400 mg/day) was started, as well as daily injections of parabulbar betamethasone during the subsequent 6 days. Microbiological analysis of the vitreous identified *Staphylococcus epidermidis*. The patient responded well to the treatment with gradual flare regression (Fig. 6). At the final follow-up visit, she had a BCVA of 0.6, a flare of 9.1 ph/ms, and no significant macular edema (Fig. 7).

## Conclusions

This study is the largest retrospective study to evaluate the utility of LFP in monitoring inflammation after intravitreal anti-VEGF injection [9, 10, 12], particularly to detect early-stage post-injection endophthalmitis. LFP is routinely used in our center to evaluate intraocular inflammation in uveitis patients and in patients undergoing intraocular procedures, namely before and after IVIs.

The two cases of bacterial endophthalmitis after 2804 injections (0.07%) at our center is in line with recent reports [3–6]. We determined the evolution of LFP flare values from pre-injection to 72 h post-injection for a total of 736 IVIs, including the 2 cases of endophthalmitis. Overall, we found that the mean LFP flare was significantly higher 72 h post-IVI than pre-IVI for patients injected with bevacizumab and aflibercept. This contrasts with previous reports [8, 9], in which no significant difference was found between mean pre and post-IVI LFP flare values after bevacizumab and ranibizumab injections, but the difference can be justified by our larger sample. Furthermore, patients treated with an IVI of aflibercept had a significantly higher 72 h post-IVI LFP flare than those injected with bevacizumab without any clinical consequences.

In order to establish a cut-off for LFP flare levels that should raise suspicion and justify a more thorough investigation, we evaluated the distribution of post-IVI LFP flare. In the two cases with flare > 50 ph/ms, there was a 3-day time span between IVI and onset of endophthalmitis, in concordance with previous reports [2, 7]. The clinical outcome was exceptionally good due to early treatment. These two cases confirm the importance of an evaluation 72-h post-IVI and show the sensitivity of using LFP to detect the early signs of endophthalmitis, even when patients are asymptomatic. Numerous reports in the literature indicate that early treatment is associated with improved visual outcomes, even with the most virulent agents [13, 14]. Follow-up with LFP also allowed verification that the improvement in inflammation was steady and gradual.

In conclusion, we propose using LFP assessment performed by an ophthalmic assistant to screen every patient 72-h post-intravitreal treatment, when such screening cannot be performed by an ophthalmologist. Flare values > 50 ph/ms should warrant immediate evaluation by an ophthalmologist.

## Abbreviations

AMD: Age-related macular degeneration
BCVA: Best corrected visual acuity
IVI: Intravitreal injection
LFP: Laser flare photometry
OCT: Optical coherence tomography
VEGF: Anti-vascular endothelial growth factor
